# Has the survival of patients with glioblastoma changed over the years?

**DOI:** 10.1038/bjc.2016.134

**Published:** 2016-05-26

**Authors:** R M deSouza, H Shaweis, C Han, V Sivasubramaniam, L Brazil, R Beaney, G Sadler, S Al-Sarraj, T Hampton, J Logan, V Hurwitz, R Bhangoo, R Gullan, K Ashkan

**Correction to:** *British Journal of Cancer* (2015) **114**, 146–150; doi:10.1038/bjc.2015.421; published online 15 December 2015

Upon publication of the above paper in the *British Journal of Cancer*, one of the authors identified an error in the spelling of their name. V Sivasubramaniam appears correctly in the author list above.

